# Supplementary material for: The mental health impact of physical inactivity: A study on UAE adolescents
Source: PLoS One. 2025 Sep 30;20(9):e0333346. doi: 10.1371/journal.pone.0333346 (PMC12483233; doi:10.1371/journal.pone.0333346)
Supplement: S1 File — (PDF) [file pone.0333346.s001.pdf]

Questionnaire

TITLE: **The Mental Health Impact of Physical Inactivity: A Study on UAE Adolescents**

I. Socio-Economic characteristics

Age	
Gender	Male 1 /Female 2
Nationality	
Grade	
Father's Education level	=< secondary/ College/ Postgraduate
Mother's Education	=< secondary/ College/ Postgraduate
Father's Occupation	
Mother's Occupation	
Number of members in the Family	
Order of the participants among siblings	1/2/3/4/=>5
Number of rooms in your house	
Weight (kg)	
Height (cm)	

General Health .II

Have you been diagnosed to have any of the following health problems:

Health problems	Yes<br>1	No<br>0
Covid-19		
Asthma		
Other respiratory problems		
Diabetes		
Heart problem		
Hypertension		
Abnormal lipid levels		
Muscle diseases		
Bone diseases		
Kidney problem		
Depression		
Other mental problems		
Other health problems		

III. Eating Habits

Frequency of eating the item/ week	0	1	2	3	4	5	6	7
Taking breakfast								
Taking fast food								
Vegetable								
Fruit								
Meat, egg								
Milk/ yogurt								
Beans, legumes								
Bread, cereal								
Processed food: Chips, sausage								

IV. Physical Activity

Please answer all the questions as honestly and accurately as you can. There are no right and wrong answers — this is not a test.

Physical activity in your spare time: Have you done any of the following activities in the past 7 days (last week)? If yes how many times? (Mark only one option per row.) .1

	No<br>0	1-2<br>1	3 - 4<br>2	5 - 6<br>3	7 times or more<br>4
Skipping					
Rowing/canoeing					
In-line skating					
Tagringette					
Walking for exercise					
Bicycling					
Jogging or running					
Aerobics					
Swimming					
Baseball, softball					
Dance					
Football					
Badminton					
Skateboarding					
Soccer					

Street hockey					
Volleyball					
Floor hockey					
Basketball					
Ice skating					
Cross-country skiing					
Ice hockey/ringette					
		Other:			

2. **In the last 7 days, during your physical education (PE) classes, how often were you very active (playing hard, running, jumping, throwing)? (Check one only.)**

- I don’t do PE **0**
- Hardly ever **1**
- Sometimes **2**
- Quite often **3**
- Always **4**

3. In the last 7 days, what did you do most of the time at recess? (Check one only.)

- Sat down (talking, reading, doing schoolwork) **0**
- Stood around or walked around **1**
- Ran or played a little bit **2**
- Ran around and played quite a bit **3**
- Ran and played hard most of the time **4**

4.In the last 7 days, what did you normally do at lunch (besides eating lunch)? (Check one only.)

- Sat down (talking, reading, doing schoolwork). **0**
- Stood around or walked around **1**
- Ran or played a little bit **2**
- Ran around and played quite a bit **3**
- Ran and played hard most of the time **4**

5.In the last 7 days, on how many days right after school, did you do sports, dance, or play games in which you were very active? (Check one only.)

- None **0**
- 1 time last week **1**
- 2 or 3 times last week **2**
- 4 times last week **3**
- 5 times last week **4**

6.In the last 7 days, on how many evenings did you do sports, dance, or play games in which you were very active? (Check one only.)

- None **0**
- 1 time last week **1**
- 2 or 3 times last week **2**
- 4- or 5-times last week **3**
- 6- or 7-times last week **4**

7.On the last weekend, how many times did you do sports, dance, or play games in which you were very active? (Check one only.)

- None **0**
- 1 time **1**
- 2— 3 times **2**
- 4 — 5 times **3**
- 6 or more times **4**

8. Which one of the following describes you best for the last 7 days?

Read all five statements before deciding on the one answer that describes you.

A. All or most of my free time was spent doing things that involve little physical effort **1**
B. I sometimes (1 — 2 times last week) did physical things in my free time (e.g. played sports, went running, swimming, bike riding, did aerobics) **2**
C. I often (3 — 4 times last week) did physical things in my free time **3**
D. I quite often (5 — 6 times last week) did physical things in my free time **4**
E. I very often (7 or more times last week) did physical things in my free time **5**

9.Mark how often you did physical activity (like playing sports, games, doing dance, or any other physical activity) for each day last week.

	**None**<br>**0**	**Little bit**<br>**1**	**Medium**<br>**2**	**Little often**<br>**3**	**Very often**<br>**4**
Monday					
Tuesday					
Wednesday					
Thursday					
Friday					
Saturday					
Sunday					

10.Were you sick last week, or did anything prevent you from doing your normal physical activities? (Check one.)

- Yes 1
- No 0

If yes, what prevented you? ______

V. Depression

Centre for Epidemiologic Studies Depression Scale (CESD)

Below is a list of the ways you might have felt or behaved. Please tell me how often you have felt this way during the past week.

1= Rarely or None of the Time (Less than 1 Day)
2= Some or a Little of the Time (1- 2 Days)
3= Occasionally or a Moderate Amount of the Time (3-4 Days)
4= Most or All of the Time (5-7 Days)

During the past week:	Rarely or None of the Time (Less than 1 Day) 0	Some or a Little of the Time (1- 2 Days) 1	Occasionally or a Moderate Amount of the Time (3-4 Days) 2	Most or All of the Time (5-7 Days) 3
1. I was bothered by things that usually don't bother me.				
2. I did not feel like eating; my appetite was poor.				
3. I felt that I could not shake off the blues even with help from my family or friends.				
4. I felt that I was just as good as other people.				
5. I had trouble keeping my mind on what I was doing.				
6. I felt depressed.				
7. I felt that everything I did was an effort.				
8. I felt hopeful about the future.				
9. I thought my life had been a failure.				
10. I felt fearful.				
11. My sleep was restless.				
12. I was happy.				

13. I talked less than usual				
14. I felt lonely.				
15. People were unfriendly.				
16. I enjoyed life.				
17. I had crying spells.				
18. I felt sad.				
19. I felt that people dislike me.				
20. I could not get going.				

Consent form

TITLE: The Mental Health Impact of Physical Inactivity: A Study on UAE Adolescents

INVESTIGATORS:

Prof. Faten Mahmoud
Prof. Shatha Al-Sharbatti
Ass Prof. Ghada Elgarawany
Abdalla Tamer Eltanbadawy
Rami Aws Alfahad

SITE(S): Al-Ameer School, Ajman; Indian school, Ajman; Al Hikma Private School Ajman; Sharjah British International School; Jumeirah English Speaking School Dubai

STUDY-RELATED PHONE NUMBER: 06 743 1333

Introduction:

We are faculty in Gulf Medical University. Your ward is being invited to participate in a research on **Prevalence and association between physical activity and mental health among adolescents in the UAE**
This Consent Form gives you information about the study we wish to conduct in the school your ward is in. You will get to know the benefits you may get if you agree to participate, how we hope to use the information that we will get from study, and who else will share the information. This will help you to make an informed decision whether your ward can participate or not.

You may choose your ward not to participate, or you may withdraw your ward's decision to Participate in the study at any time. In either case, your ward will not lose any benefits to which your ward is otherwise entitled.

Purpose of the research

Adolescence period is a very important and critical stage in the human evolution process. A lot of physiological and social factors that can increase the prevalence of mental problems during this period. Therefore, it is important to know **the prevalence and association between physical activity and mental health**. The main aim of this research is

a. to assess the prevalence of physical inactivity and depression
b. to assess the association between physical inactivity and depression
c. To assess factors associated with physical inactivity and depression

We are targeting high school students as it is a transition period of a person's life in many ways.

Participant selection and number of participants

The study will include students Grade 9-12 who will be selected from the following Schools: Al-Ameer School, Ajman; Indian school, Ajman; Al Hikma Private School Ajman; Sharjah British International School; Jumeirah English Speaking School Dubai. Approximately 271 individuals will take part in this research.

Why am I being asked to participate in this study?

Your ward is asked to participate in this research because your ward fulfils the inclusion criteria

How long will I be in this study?

Your ward will be in this study for 10-15 minutes that are needed for filling a questionnaire

What will I be asked to do?

Your ward will be asked to fill a questionnaire that include; socio-demography, information on health, lifestyle and psychological related questions.

What benefit can I expect from participating in the study?

There is no direct benefit, However, participation of your son / daughter will help us to write recommendations that may help in the control of this problem.

Confidentiality, Anonymity and storage of data

We assure you that the study is anonymous, the information your ward is going to provide will be analysed by the group and there will be no link between you as a person and the results. Confidentiality of the information will be respected and only the research team, and Ethics Committee member in GMU may have access to the data**.** The data will be stored for 3 years by the principle investigator

Provision of feedback to the participant

Results of this study will be published/ presented in national / international conferences.

Provision made for distribution of copy of the consent form to all participants

If you accept you ward to participate in this study you will be provided with copy of this informed consent

Can I participate in this study if I do not sign this consent form?

To participate in this research you will have to sign this informed consent, then your ward will be provided with copy of the same

Signature of Consent

I have read this Consent Form. All my questions have been answered. I allow my ward to take part in this study. I authorise the use and disclosure of my ward's questionnaire responses to the parties listed in the authorization section of this consent for the purposes described above.

Signature of Subject's parent (below the age of 18) /subject with Date:

Signature of the interviewer with name and date:
